# Cancer-associated fibroblasts induce sorafenib resistance of hepatocellular carcinoma cells through CXCL12/FOLR1

**DOI:** 10.1186/s12885-023-11613-8

**Published:** 2023-12-06

**Authors:** Jiali Zhao, En Lin, Zirui Bai, Yingbin Jia, Bo Wang, Yihua Dai, Wenfeng Zhuo, Guifang Zeng, Xialei Liu, Chaonong Cai, Peiping Li, Baojia Zou, Jian Li

**Affiliations:** 1 Department of Hepatobiliary Surgery, Fifth Affiliated Hospital of Sun Yat-Sen University, Zhuhai, 519000 Guangdong China; 2 Department of Urology Surgery, Fifth Affiliated Hospital of Sun Yat-Sen University, Zhuhai, 519000 Guangdong China; 3 Department of Anesthesiology, Fifth Affiliated Hospital of Sun Yat-Sen University, Zhuhai, 519000 Guangdong China

**Keywords:** Cancer-associated fibroblasts, Hepatocellular carcinoma, Sorafenib, Resistance

## Abstract

**Background:**

Due to the high drug resistance of hepatocellular carcinoma (HCC), sorafenib has limited efficacy in the treatment of advanced HCC. Cancer-associated fibroblasts (CAFs) play an important regulatory role in the induction of chemoresistance. This study aimed to clarify the mechanism underlying CAF-mediated resistance to sorafenib in HCC.

**Methods:**

Immunohistochemistry and immunofluorescence showed that the activation of CAFs was enhanced in HCC tissues. CAFs and paracancerous normal fibroblasts (NFs) were isolated from the cancer and paracancerous tissues of HCC, respectively. Cell cloning assays, ELISAs, and flow cytometry were used to detect whether CAFs induced sorafenib resistance in HCC cells via CXCL12. Western blotting and qPCR showed that CXCL12 induces sorafenib resistance in HCC cells by upregulating FOLR1. We investigated whether FOLR1 was the target molecule of CAFs regulating sorafenib resistance in HCC cells by querying gene expression data for human HCC specimens from the GEO database.

**Results:**

High levels of activated CAFs were present in HCC tissues but not in paracancerous tissues. CAFs decreased the sensitivity of HCC cells to sorafenib. We found that CAFs secrete CXCL12, which upregulates FOLR1 in HCC cells to induce sorafenib resistance.

**Conclusions:**

CAFs induce sorafenib resistance in HCC cells through CXCL12/FOLR1.

**Supplementary Information:**

The online version contains supplementary material available at 10.1186/s12885-023-11613-8.

## Background

Hepatocellular carcinoma (HCC) is one of the most malignant tumours worldwide, and the incidence is increasing [1]. Studies have shown that more than 50% of HCC patients are initially diagnosed at an advanced stage [2]. Sorafenib is the first Food and Drug Administration (FDA)-approved targeted therapy for advanced HCC [3]. However, since most advanced HCC patients acquire resistance to sorafenib, only 10% to 30% of patients show objective responses to sorafenib [2]. Therefore, the prognosis of advanced HCC patients remains poor, even after sorafenib treatment [4, 5]. Thus, we need to further investigate the mechanisms of sorafenib resistance in HCC. It is important to develop novel therapeutic strategies to overcome sorafenib resistance in patients with advanced HCC.

Cancer-associated fibroblasts (CAFs) represent the main cell population of the tumour microenvironment (TME) [6]. CAFs can stimulate tumour cell drug resistance, proliferation, invasion, and metastasis in various cancers, including HCC [7, 8]. Recent studies have shown that CAFs play a crucial role in the induction of chemoresistance in a variety of cancers, including HCC [9–12]. However, the mechanism of CAF-mediated sorafenib resistance in HCC remains to be fully elucidated. Clarifying this mechanism is a crucial step towards identifying novel therapeutic targets to overcome chemotherapy resistance and predicting treatment response.

Therefore, we aimed to investigate the mechanism of CAF-induced sorafenib resistance in HCC. This study can help us to identify novel therapeutic targets for overcoming sorafenib resistance. It will be significant to reduce the rate of sorafenib resistance and improve the prognosis of advanced HCC patients.

## Methods

### Isolation of fibroblasts from cancer tissues and paracancerous tissues in HCC samples

Human HCC tissues were obtained in post-surgical samples, from the department of hepatobiliary surgery, at the Fifth Affiliated Hospital of Sun Yat-sen University. All subjects signed an informed consent form, and the study was approved by the Ethics Committee of the Fifth Affiliated Hospital of Sun Yat-sen University.

Cancer-associated fibroblasts (CAFs) and paracancerous normal fibroblasts (NFs) were isolated from HCC tissues and non-tumor tissues adjacent to the HCC, respectively. Fresh HCC tissues and paracancerous tissues were washed with phosphate buffer (PBS; GenDEPOT, Barker, TX, USA) and mince into small pieces (< 1 mm^3^). Five minuted small tissues were attached to the cell culture dishes and treated with Dulbecco's modified eagle medium (DMEM medium, Gibco, USA), containing 10% fetal bovine serum (FBS; Invitrogen, Waltham, MA, USA), 50 U/mol penicillin (Sigma-Aldrich, USA) and 50 mg/ml streptomycin (Sigma-Aldrich, USA). The DMEM medium was changed every two days. The fibroblasts extending from the HCC tissue were then trypsinised and transferred to the dish, followed by incubation in fresh medium to facilitate attachment of the isolated fibroblasts to the dish. Cells were maintained in complete medium at 37° C in a humidified incubator with 5% CO_2_ and 21% O_2_.

### Cell lines

HCC cell lines (HepG2, Huh7) were obtained from the Cancer Center, Sun Yat-sen University, Guangzhou, Guangdong, People’s Republic of China. Cancer-associated fibroblasts (CAFs) and normal fibroblasts (NFs) were cultured from postoperative HCC tissues in our center. All cells were cultured in Dulbecco’s modified eagle medium (DMEM medium, Gibco, USA), which was supplemented with 10% fetal bovine serum (FBS), 50 U/mol penicillin (Sigma-Aldrich, USA) and 50 mg/ml streptomycin (Sigma-Aldrich, USA). AMD3100, an inhibitor of CXCL12, was purchased from Selleck (S8030). CXCL12 protein was purchased from MedChemExpress (HY-P7287). The anti-CXCR4 was purchased from Proteintech (60042–1-Ig). All cultured cells were kept in a humidified incubator at the temperature of 37°C, under the concentration of 5% CO_2_, and 21% O_2_.

### Cell transfection

For the transfection of human hepatic carcinoma cells (Huh7, HepG2), lentivirus with scramble (control) and sh-CXCR4 was used in the experiments, which was constructed and purchased from Genechem (Shanghai, China). Fourty-eight hours after transfection, stable cell lines were screened by DMEM (10% FBS) containing 2ug/ml puromycin. Western blot analyses were performed to verify the success of transfection.

### HCC cells were cocultured with CAFs and NFs

CAFs (1 × 10^6^ cells/ml) or NFs (1 × 10^6^ cells /ml) were cultured in Dulbecco’s modified eagle medium (DMEM medium, Gibco, USA), which were supplemented with 10% fetal bovine serum (FBS) for 48 h. Then, their supernatant was collected to treat HCC cells (HepG2 and Huh7) for 48 h.

### Western blotting analyses

After all sample proteins were separated by SDS-PAGE gel, they were transferred to the PVDF membrane and then blocked with 5% skimmed milk. Then, they were subsequently incubated with primary and secondary antibodies. The primary antibodies used to detect the target protein were β-actin (abclonal, AC026, 1:1000), FOLR1 (Proteintech, 23355–1-AP, 1:1000), CXCR4 (Proteintech, 60042–1-Ig, 1:1000), Cleaved Caspase-3 (Cell Signaling Technology, 5A1E, 1:500). The targeted bands were analyzed by ImageJ software (v1.8.0; National Institutes of Health, USA). The β-actin was used as the internal control. The relative protein levels were quantified through comparison to β-actin.

### Cell viability assays

Cell proliferation was analyzed by the cell counting kit-8 (CCK-8, MedChemExpress, Cat. No. HY-K0301). Cells were seeded at a density of 1 × 10^4^ /well into 96-well microplates. Then, the cells were treated with various concentrations of Sorafenib (0.25, 0.5, 1, 2, 4, 8, 16, and 32 μM). The CCK-8 assay was performed after 48 h of treatment. Treated cells were incubated for 4 h with a culture medium containing the CCK-8 reagent, and absorbance was recorded at 450 nm using the iMark™ Microplate Absorbance Reader (Bio-Rad, iMark, United States). All experiments were repeated three times. The inhibition of cell proliferation was expressed by the absorbance.

### Flow cytometry apoptosis assay

Cell apoptosis analysis: cells were implanted into a 6-well plate for apoptosis analysis. Then, the medium was replaced with a fresh medium supplemented with 3 μM Sorafenib. After treatment of 48 h, the cell apoptosis was detected by Apoptosis Detection Kit (eBioscience™ Annexin V Apoptosis Detection Kits, Thermo Fisher Scientific, Lot No. 2106736). All cells were detected by Polychromatic analytical flow cytometry (Beckman, Cytoflex LX, United States).

### Colony formation assay

HCC cells (800 cells/well) were implanted into a 6-well plate. Then, they were treated with the supernatant of CAFs or NFs, CXCL12 protein, AMD3100 (20 μM), and sorafenib (3 μM). After treatment of 15 days, cells were fixed with 4% paraformaldehyde and stained with crystal violet (5%). Each experiment was done thrice. Cells colonies formation rate = Number of colonies formed in each treatment group / Number of implanted cells (800 cells) × 100%.

### Enzyme-linked immunosorbent assay (Elisa)

The supernatants of CAFs and NFs were carried out with ELISA analyses. The OD value was detected with the enzyme plate analyzer. Meanwhile, the amount of target protein secreted per 100,000 cells was calculated. The Elisa kit was a Human stromal cell-derived factor 1β (CXCL12β/SDF1B) ELISA kit (Cusabio, B04011121).

### Immunohistochemistry staining (IHC)

Human HCC tissues and the xenograft tumors of mice were sectioned into 5 μm slices. Hematoxylin and eosin staining was applied to confirm the status of cancer or cancer-free. All tissue sections were baked, dehydrated, hydrated, and antigen-retrieved. Then, they were incubated with primary and secondary antibodies. An N-ACHROPLAN microscope (ZEISS, Germany) was used to photograph the representative areas. Image-Pro Plus v6.0 software (Media Cybernetics Inc., Bethesda, MD, USA) was used to analyze the Information Object Definition (IOD) values of all images. We calculated the relative IOD values based on three parameters (sum of area, average density, and IOD), which were used for further analysis.

IHC was performed according to the kits (Boster, SA1028, SA1027). Primary antibodies were used for IHC staining: α-SMA (Abcam, ab119952, 1:100), Cleaved Caspase-3 (Cell Signaling Technology, 5A1E, 1:100).

### Immunohistofluorescence staining (IF)

Human HCC tissue samples were sectioned into 5 μm slices. Hematoxylin and eosin staining was applied to confirm the status of cancer or cancer-free. IF was performed according to the kits (Boster, SA1028, SA1027). Primary antibodies were used for IHC staining: CXCL12 (Boster, BA1389, 1:100), and α-SMA (Abcam, ab119952, 1:100).

### Animal xenograft models

The growth of the tumor was observed in vivo. A total of 9 BALB/c mice (4 weeks old; 9 males) were purchased from Guangdong Medical Laboratory Animal Center (Guangdong, China) and housed with a 12 h light/dark cycle and fed standard laboratory food and water. We randomly divided the 9 mice into the NC group, CAFs group, and CAFs + AMD3100 group equally, with 3 mice in each group.

1 × 10^6^ HepG2 cells (50 μL) and 1 × 10^6^ NFs cells (50 μL) were mixed and then injected subcutaneously into the left and right flanks of nude mice in the NC group. Meanwhile, 1 × 10^6^ HepG2 cells (50 μL) and 1 × 10^6^ CAFs (50 μL) were mixed and then injected subcutaneously into the left and right flanks of nude mice in the CAFs group, and CAFs + AMD3100 group. Therefore, we would get 6 tumors in each group. The tumor volume (mm^3^) = (length of tumor × width of tumor^2^)/2. When tumor volume reached 100 – 150 mm^3^, the mouse of the NC group and CAFs group were began to receive tail vein injections of an equal volume of normal saline + Sorafenib (30 mg/kg) for 3 weeks. Meanwhile, the mouse of the CAFs + AMD3100 group was began to receive tail vein injections of an equal volume of AMD3100 (2.5 mg/kg) + Sorafenib (30 mg/kg) for 3 weeks.

### Statistical analysis

Statistical analysis was performed using GraphPad Prism V.8 software. All data were repeated at least three times. Data were presented as mean ± SEM and analyzed by Student’s t-test and Pearson correlation. One-way analysis of variance (ANOVA) and Brown-Forsythe tests were carried out for multiple group comparisons. Kaplan-Miere analysis and Log-rank test as used to analyze the survival of HCC patients. For each test, values of *p* < 0.05 were considered statistically significant. *p < 0.01; **p < 0.001; ***p < 0.0001; ****p < 0.00001. N.S, not significance.

## Results

### CAFs were increasingly activated in HCC tissues, compared with paracancerous tissues

**Fig. 1:**
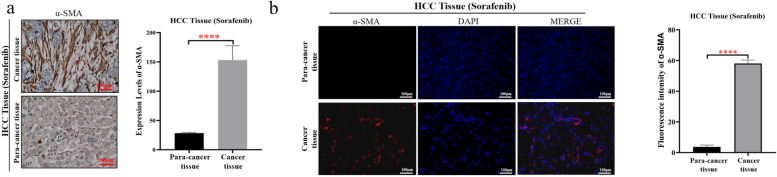
CAFs were significantly activated in cancer tissues compared with paracancerous tissues in HCC samples. **a** Representative images of fibroblasts with the expression of α-SMA in HCC tissues and paracancerous tissues (left). Statistical plot of fibroblasts expressing α-SMA in HCC tissues and paracancerous tissues (right). **b** Immunofluorescence representative images: the expression of α-SMA in fibroblasts in HCC tissues and paracancerous tissues (left). Statistical plot of fluorescence intensity of fibroblasts expressing α-SMA in HCC tissues and paracancerous tissues (right). The data presented mean ± SEM. ****p < 0.00001

α-SMA is a common marker of CAF activation [13, 14]. The results of IHC and IF revealed that the activation of CAFs was significantly increased in HCC cancer tissues, compared with paratumour tissues (Fig. 1a and b).

### CAFs induced sorafenib resistance in HCC cells

**Fig. 2:**
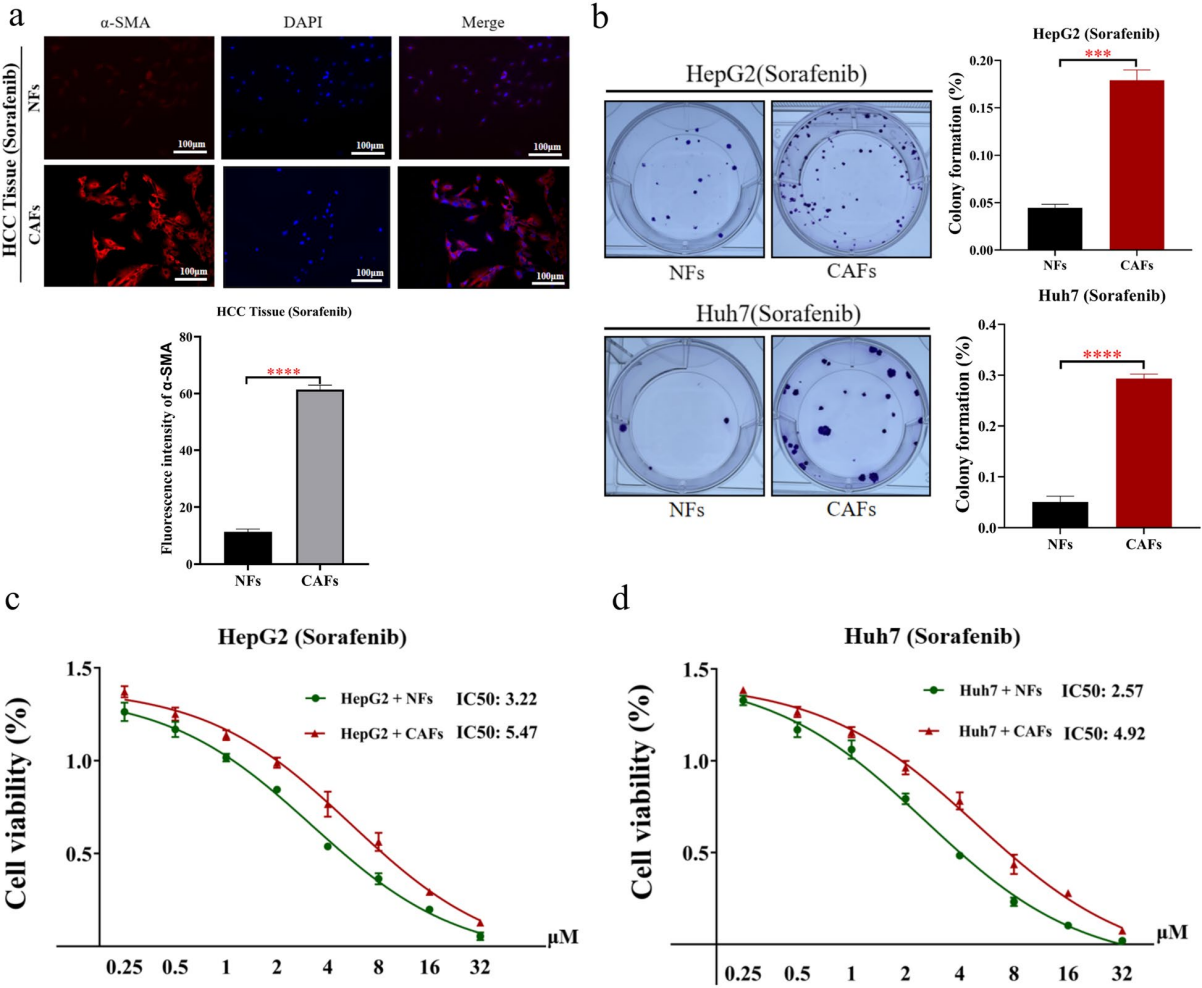
Compared with NFs, CAFs significantly promoted sorafenib resistance in HCC cells. **a** CAFs were isolated from HCC tissues. Meanwhile, paracancerous normal fibroblasts (NFs) were isolated from non-tumor tissues adjacent to HCC (upper). Statistical plot of fluorescence intensity of fibroblasts expressing α-SMA in HCC tissues and paracancerous tissues (below). **b** Colony forming assays of HCC cells (HepG2 and Huh7), which were treated with the cellular supernatant of CAFs and NFs, and sorafenib. **c**,** d** Cell viability assay: compared with NFs, CAFs significantly enhanced sorafenib resistance in HCC cells (HepG2 and Huh7). The data presented mean ± SEM. ***p < 0.0001; ****p < 0.00001

Then, we sorted CAFs from cancer tissues in HCC (Fig. 2a). Meanwhile, we sorted NFs from paracancerous tissues in HCC (Fig. 2a). To explore whether CAFs can induce sorafenib resistance in HCC cells, we cocultured CAFs, NFs and HCC cells. The results showed that CAFs significantly enhanced the resistance of HCC cells to sorafenib (Fig. 2b-d). Therefore, CAFs were significantly activated in cancer tissues of HCC, which suppressed the sensitivity of HCC cells to sorafenib.

### CAFs secrete CXCL12 to induce sorafenib resistance in HCC cells

**Fig. 3:**
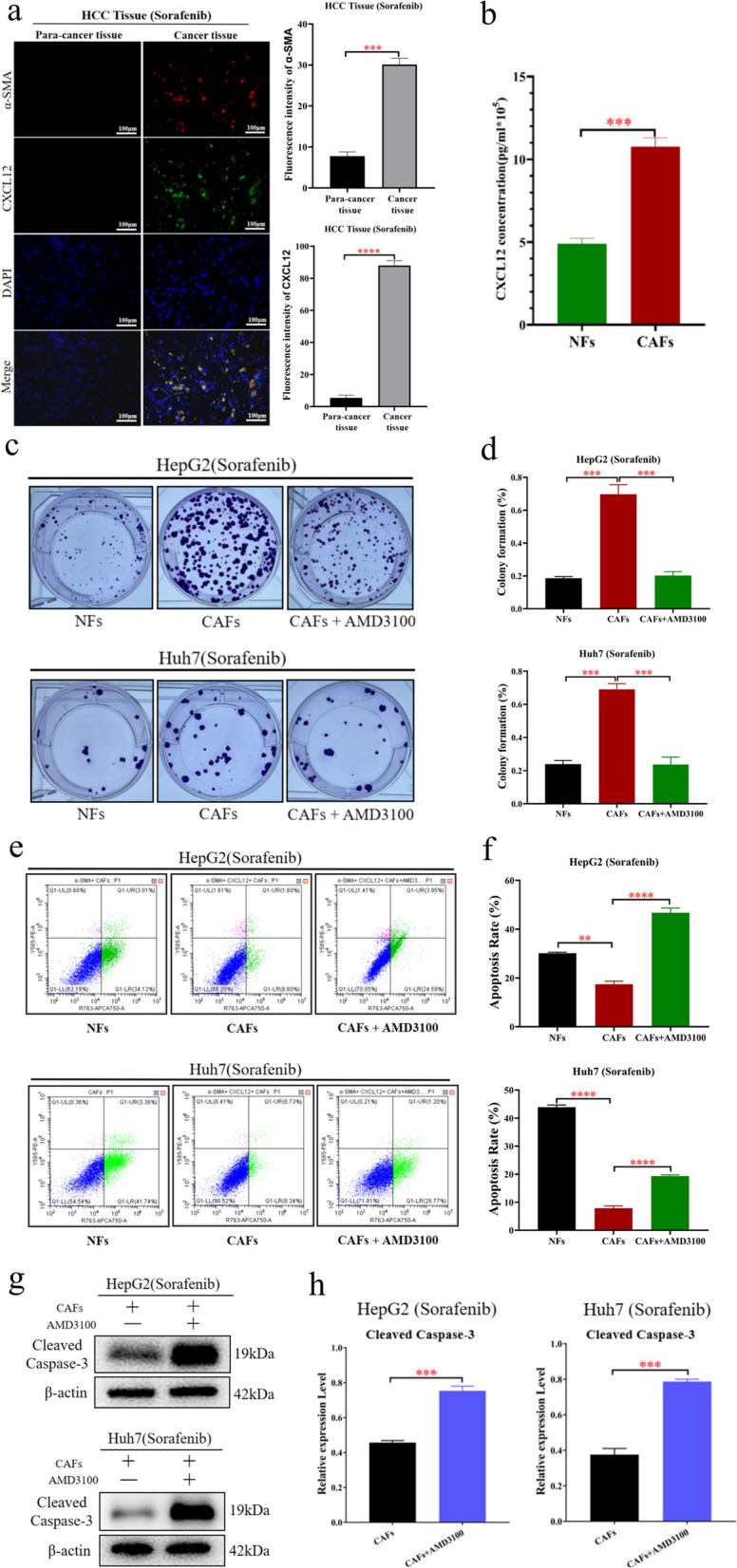
CAFs induce sorafenib resistance in HCC cells by secreting CXCL12. **a** The results of immunofluorescence showed that the expression of CXCL12 in CAFs in HCC tissues was significantly higher than that in paracancerous tissues (left). Statistical plot of fluorescence intensity of fibroblasts expressing α-SMA and CXCL12 in HCC tissues and paracancerous tissues (right). **b** ELISA showed that CAFs secreted higher level of CXCL12 than NFs. **c**, **d** Colony forming assays detected the sorafenib resistance of HCC cells (HepG2 and Huh7), after treated with the cellular supernatant of CAFs and NFs, sorafenib, and AMD3100. **e**, **f** Flow cytometry apoptosis assay detected the sorafenib resistance of HCC cells (HepG2 and Huh7), after treated with the cellular supernatant of CAFs and NFs, sorafenib, and AMD3100. **g**, **h** Western blotting was performed to detect the expression of β-actin, and Cleaved Caspase-3 in HCC cells (HepG2 and Huh7), which were treated with the cellular supernatant of CAFs, sorafenib, and AMD3100. The data presented mean ± SEM. *p < 0.01; **p < 0.001; ***p < 0.0001; ****p < 0.00001

CXCL12 is a common secreted factor in CAFs [15]. Studies have shown that CXCL12 can induce drug resistance in cancer [16–18]. However, there is no report on whether CXCL12 can induce sorafenib resistance in HCC cells. To explore whether CAFs secrete CXCL12 to induce sorafenib resistance in HCC cells, the following experiments were performed. Immunofluorescence assays showed that, CAFs in HCC tissues expressed higher levels of CXCL12 than NFs in paracancerous tissues (Fig. 3a). The ELISA results showed that CAFs secreted higher levels of CXCL12 than NFs (Fig. 3b). Moreover, the results of cell cloning assays, and flow cytometry apoptosis analysis showed that CAFs significantly reduced the sensitivity of HCC cells to sorafenib (Fig. 3c-f). When CXCL12 was inhibited, the sensitivity of HCC cells to sorafenib was increased (Fig. 3c-h). These results suggest that CAFs induce sorafenib resistance in HCC cells through CXCL12.

### CXCL12 induces sorafenib resistance in HCC cells by upregulating FOLR1

**Fig. 4:**
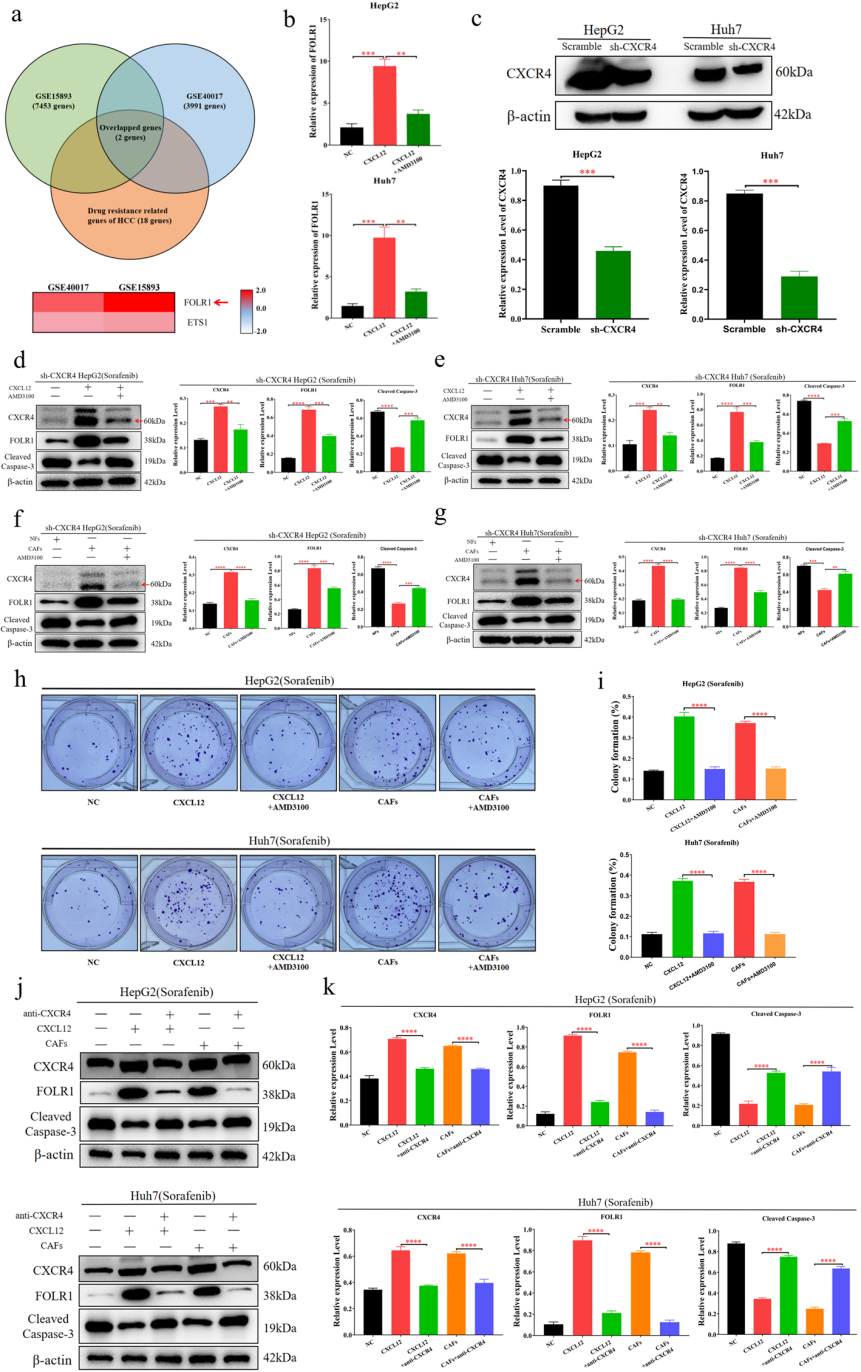
CXCL12 induces sorafenib resistance in HCC cells by up-regulating the expression of FOLR1. **a** We found two datasets of cancer cells treated with CXCL12 protein (GSE15893 and GSE40017) in the GEO database. We took the intersection of the differentially expressed genes between these two datasets and combined them with the reported drug-resistant genes of HCC to obtain two genes. FOLR1 was the most significantly upregulated drug resistance-related gene upon CXCL12 treatment. **b** The qPCR was performed to detect the level of FOLR1 in Huh7 and HepG2, which treated with CXCL12 protein and AMD3100. **c** Western blotting was performed to detect the expression of β-actin and CXCR4 in CXCR4 knockdown HCC cells (Huh7 and HepG2). **d**, **e** Western blotting was performed to detect the expression of β-actin, CXCR4, FOLR1, and Cleaved Caspase-3 in Huh7 and HepG2, after treated with sorafenib, CXCL12 protein, and AMD3100. **f**, **g** Western blotting was performed to detect the expression of β-actin, CXCR4, FOLR1, and Cleaved Caspase-3 in Huh7 and HepG2, after treated with sorafenib, AMD3100, the supernatant of CAFs, and NFs. **h**, **i** Colony forming assay detected the sorafenib resistance of HCC cells (HepG2 and Huh7), after treated with sorafenib, CXCL12 protein, the supernatant of CAFs, and AMD3100. **j**, **k** Western blotting was performed to detect the expression of β-actin, FOLR1, CXCR4, and Cleaved Caspase-3 in Huh7 and HepG2, after treated with sorafenib, anti-CXCR4, CXCL12 protein, and the supernatant of CAFs. The data presented mean ± SEM. **p < 0.001; ***p < 0.0001; ****p < 0.00001

To explore the mechanism by which CXCL12 secreted by CAFs induces sorafenib resistance in HCC cells, we assessed two datasets of cancer cells treated with CXCL12 protein (GSE15893 and GSE40017) in the GEO database. We took the intersection of the differentially expressed genes between these two datasets and combined them with the reported drug resistance genes of HCC [2, 19, 20]. The results showed that FOLR1 was the most significantly upregulated drug resistance-related gene upon CXCL12 treatment (Fig. 4a). Subsequently, we used CXCL12 protein to treat HCC cells. The qPCR results showed that CXCL12 protein significantly upregulated FOLR1 at the mRNA level in HCC cells (Fig. 4b). To further clarify the role of CXCL12 in HCC cells, the CXCR4 knockdown HCC cell lines (HepG2 and Huh7) were constructed (Fig. 4c). The results showed that CXCL12 protein upregulated FOLR1 in HCC cells, and CAFs significantly increased secretion of FOLR1 in HCC cells; both of these phenotypes were associated with sorafenib resistance in HCC cells (Fig. 4d-i). Moreover, when CXCL2 was inhibited by AMD3100, these effects disappeared (Fig. 4d-i).

Studies have revealed that the upregulation of FOLR1 is associated with sorafenib resistance in cancer cells [19, 21]. Additionally, CXCR4 has been identified as the major cognate receptor for CXCL12. The CXCL12/CXCR4 biological axis plays an important role in the malignant progression of cancer [17, 22, 23]. To investigate whether CXCL12 upregulates FOLR1 to induce sorafenib resistance in HCC cells through CXCR4, we inhibited CXCR4 in HCC cells and subsequently treated them with CXCL12 protein or CAF supernatant. The results demonstrated that when CXCR4 was inhibited, the ability of CXCL12 protein or CAF supernatant to upregulate FOLR1 in HCC cells was inhibited, and the sensitivity of HCC cells to sorafenib was increased (Fig. 4j-k). These findings suggest that CAFs induce sorafenib resistance in HCC cells through the CXCL12/CXCR4/FOLR1 pathway.

### CAFs enhance sorafenib resistance in HCC cells through CXCL12 in vivo

**Fig. 5:**
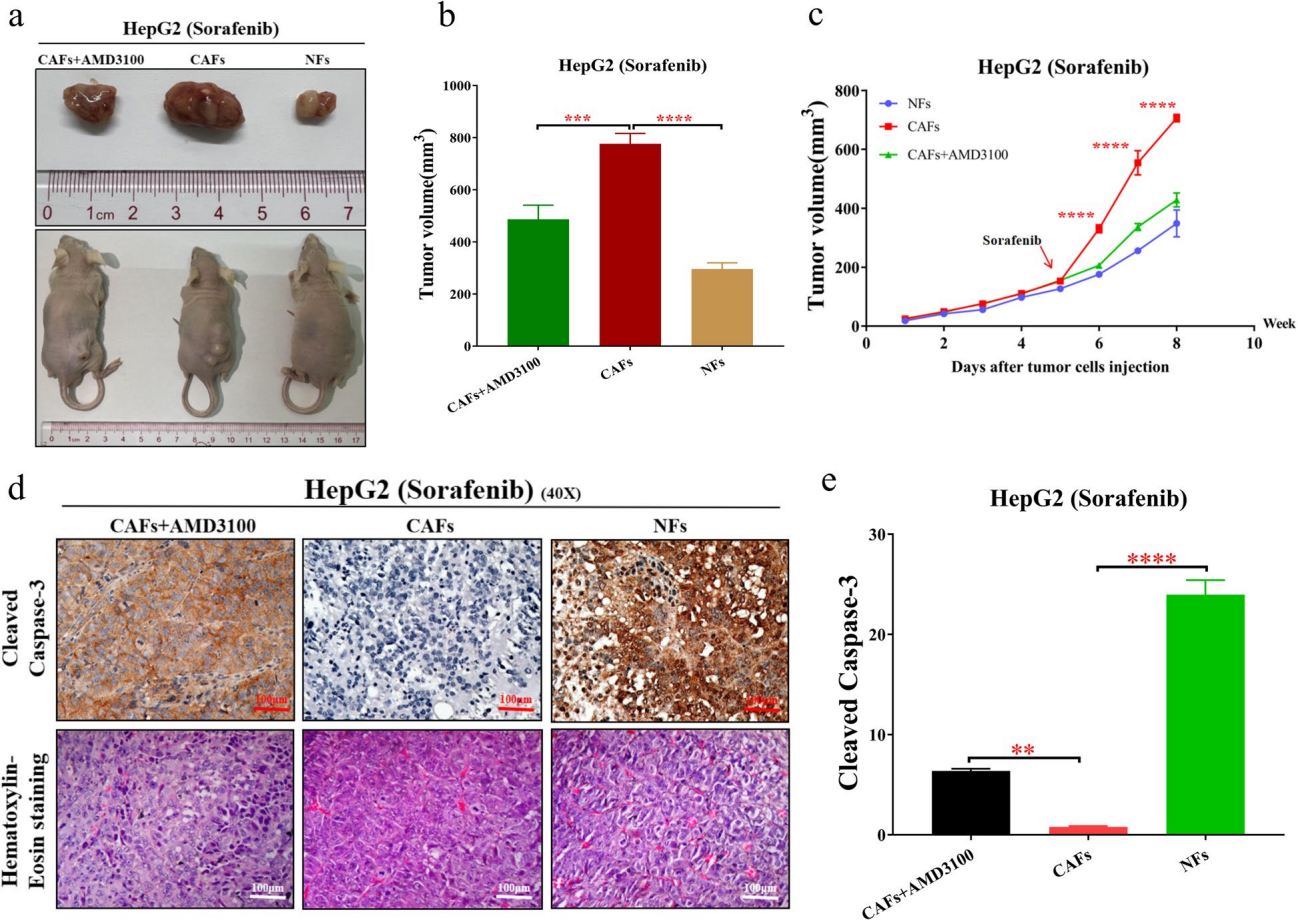
CAFs enhance sorafenib resistance of HCC cells through CXCL12 in vivo. **a** Representative images of tumors in mice of CAFs + AMD3100 group, CAFs group, and NFs group after different treatments. **b** The tumor volume in different treatment groups. **c** The tumor proliferation trend in different treatment groups. **d** Pathological validation of tumors under a microscope (40X), after H&E staining and Immunohistochemistry in tumor tissues. The immunohistochemistry staining to detect the expression of Cleaved Caspase-3 in different treatment groups from the tumor tissues of mice. **e** The expression level of Cleaved Caspase-3 in different treatment groups from the tumors of mice. The data presented mean ± SEM. **p < 0.001; ***p < 0.0001; ****p < 0.00001

Next, to further verify the effects of CAF-induced sorafenib resistance in HCC cells in vivo, a xenograft model in nude mice was established (Fig. 5a). The results showed that CAFs significantly enhanced the resistance of HCC cells to sorafenib, while CXCL12 inhibition reversed this phenomenon (Fig. 5b-c). Furthermore, the IHC results showed that during sorafenib treatment, CAFs reduced the sensitivity of HCC cells to sorafenib, while CXCL12 inhibition promoted the apoptosis of HCC cells (Fig. 5d-e). In conclusion, CAFs could induce sorafenib resistance in HCC cells via CXCL12 in vivo.

## Discussion

Sorafenib is a first-line targeted drug for the treatment of advanced HCC, but increasing evidence has shown that the high drug resistance of HCC cells leads to poor efficacy of sorafenib [2]. Various groups have attempted to investigate the underlying mechanisms of sorafenib resistance in HCC and to identify new therapeutic targets to overcome drug resistance [24, 25]. In this study, we determined that the activation level of CAFs is significantly increased in HCC tissues, which could induce sorafenib resistance in HCC cells. Moreover, CAFs secrete C-X-C motif chemokine 12 (CXCL12) to upregulate FOLR1 expression in HCC cells via the CXCL12/CXCR4 axis. Furthermore, the overexpression of FOLR1 is positively correlated with sorafenib resistance in HCC. Finally, our results revealed that CAFs induce sorafenib resistance in HCC cells through the CXCL12/CXCR4/FOLR1 pathway.

Generally, CAFs promote the malignant progression of cancer cells by secreting cytokines, chemokines, and proangiogenic factors, including CXCL12, TGF-β1, VEGF, PDCF, IL-6 and CXCL16 [26–28]. Among them, SDF-1 and TGF-β1 are two of the most-powerful and widely investigated molecules in various solid tumours, including HCC, pancreatic carcinoma, and colorectal cancer [26–28]. Our results also suggest that compared with NFs, CAFs secrete higher levels of CXCL12, which specifically binds to CXCR4 in HCC cells.

CXCL12, also known as stromal cell-derived factor-1 (SDF-1), is an extracellular homeostatic chemokine that often binds to CXCR4, which could regulate cancer cell malignant progression [29–31]. Relevant studies have shown that the high expression of CXCL12 could promote drug resistance in pancreatic cancer, breast cancer, and acute lymphoblastic leukaemia [16–18]. However, the underlying mechanism by which CXCL12 regulates sorafenib resistance in HCC cells remains unclear. In this study, we found that CXCL12, secreted by CAFs, induced sorafenib resistance in HCC cells by upregulating FOLR1.

Folate receptor 1 (FOLR1), a protein receptor for transporting folate into cells, is expressed at a very low level in normal epithelial cells [19, 32–35], but is abnormally upregulated in HCC, ovarian cancer, and pancreatic cancer [19, 32–35]. Studies have found that FOLR1 plays a crucial role in various types of malignant cancers [19, 21, 36, 37]. Furthermore, the upregulation of FOLR1 was correlated with the drug resistance of cancer cells [19]. However, the mechanism of FOLR1 upregulation in HCC cells is still unclear, especially in the tumour environment. As reported in our study, we found that FOLR1 is upregulated by CAF-secreted CXCL12. These results suggest that CAFs secrete CXCL12 to induce sorafenib resistance in HCC cells by upregulating FOLR1. However, further research is needed to explore the molecular mechanism of the effect of FOLR1 on cancer cell behaviour.

Sorafenib resistance is a persistent clinical challenge for HCC therapy. CAFs act as a key player in HCC cell evasion of chemotherapy drugs [11, 38]. In this study, we revealed that CAFs secrete CXCL12 to induce sorafenib resistance in HCC cells. CXCL12 has been revealed to participate in the development of chemotherapy resistance in various tumours [18, 22, 39]. However, there is no study on the correlation between CXCL12 and FOLR1 in sorafenib resistant HCC. Our study showed that CAFs secreted CXCL12 to induce sorafenib resistance in HCC cells by upregulating FOLR1.

## Conclusion

In summary, we demonstrated that the activation level of CAFs is increased in HCC tissues, which significantly enhances sorafenib resistance in HCC cells. CAFs secrete CXCL12 to induce sorafenib resistance in HCC cells by upregulating FOLR1. Our study provides the first evidence that the CXCL12/CXCR4/FOLR1 axis is associated with sorafenib resistance in HCC, suggesting a potential new target for improving the efficacy of sorafenib and the prognosis of HCC patients.

## Supplementary Information

**Additional file 1.**

## Data Availability

All data generated or analyzed during this study are included in this published article and are also available from the corresponding author on reasonable request.

## Abbreviations

HCC: Hepatocellular carcinoma
CAFs: Cancer-associated fibroblasts
α-SMA: α-Smooth muscle actin
CXCL12: Stromal cell-derived factor-1 (SDF-1)
CXCR4: CXC receptor 4
FOLR1: Folate receptor 1
JAK: Janus kinase
STAT3: Signal transducer and activator of transcription 3
ERK1/2: Extracellular signal-related kinases 1 and 2
BAFF: B cell activating factor
NF-κB: Nuclear factor-κB
IL6: Interleukin-6
IL17A: Interluekin-17A
IGF1: Insulin-like growth factor 1
ChIP-qPCR: Chromatin immunoprecipitation quantitative PCR assays
SHH: Sonic Hedgehog
FAP: Fibroblast activation protein
SPP1: Plasma-secreted phosphoprotein 1
IKKβ: Inhibitor of nuclear factor kappa-B kinase subunit beta
SULF2: Sulfatase 2
GEO: Gene Expression Omnibus
