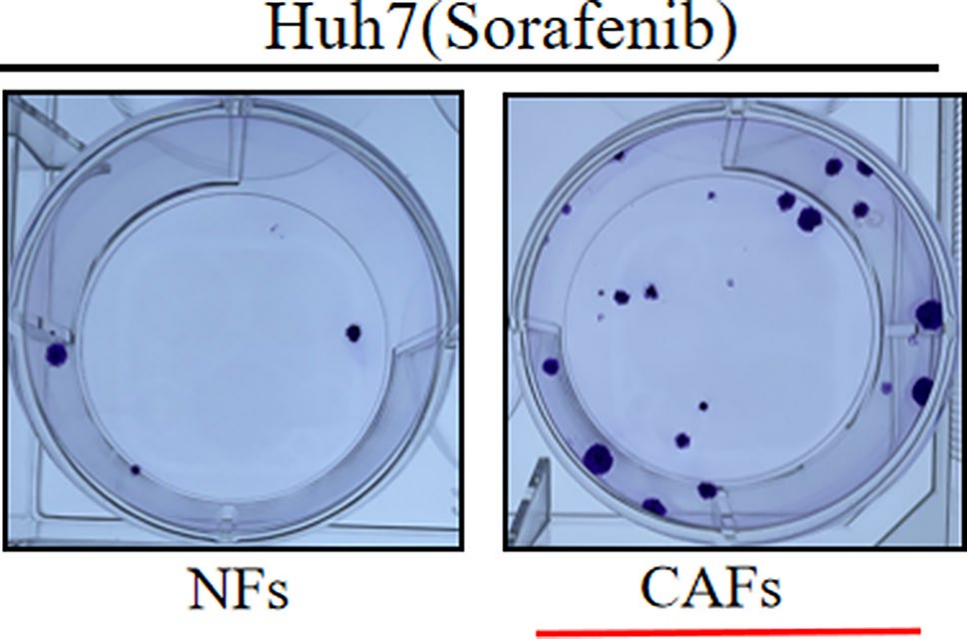# Correction: Cancer-associated fibroblasts induce sorafenib resistance of hepatocellular carcinoma cells through CXCL12/FOLR1

**DOI:** 10.1186/s12885-025-13807-8

**Published:** 2025-03-04

**Authors:** Jiali Zhao, En Lin, Zirui Bai, Yingbin Jia, Bo Wang, Yihua Dai, Wenfeng Zhuo, Guifang Zeng, Xialei Liu, Chaonong Cai, Peiping Li, Baojia Zou, Jian Li

**Affiliations:** 1 Department of Hepatobiliary Surgery, Fifth Affiliated Hospital of Sun Yat-Sen University, Zhuhai, Guangdong 519000 China; 2 Department of Urology Surgery, Fifth Affiliated Hospital of Sun Yat-Sen University, Zhuhai, Guangdong 519000 China; 3 Department of Anesthesiology, Fifth Affiliated Hospital of Sun Yat-Sen University, Zhuhai, Guangdong 519000 China

**Correction: BMC Cancer 23, 1198 (2023)**

10.1186/s12885-023-11613-8

Following publication of the original article [1], the authors identified that the image of “Huh7 (Sorafenib) CAFs” group in Figure 2b of this article is wrong, which is the same as the image of “Huh7 (Sorafenib) CAFs + AMD3100” in Figure 3c.

In fact, the image of “Huh7 (Sorafenib) CAFs” (Figure 2b) belongs to “Huh7 (Sorafenib) CAFs + AMD3100” (Figure 3c).


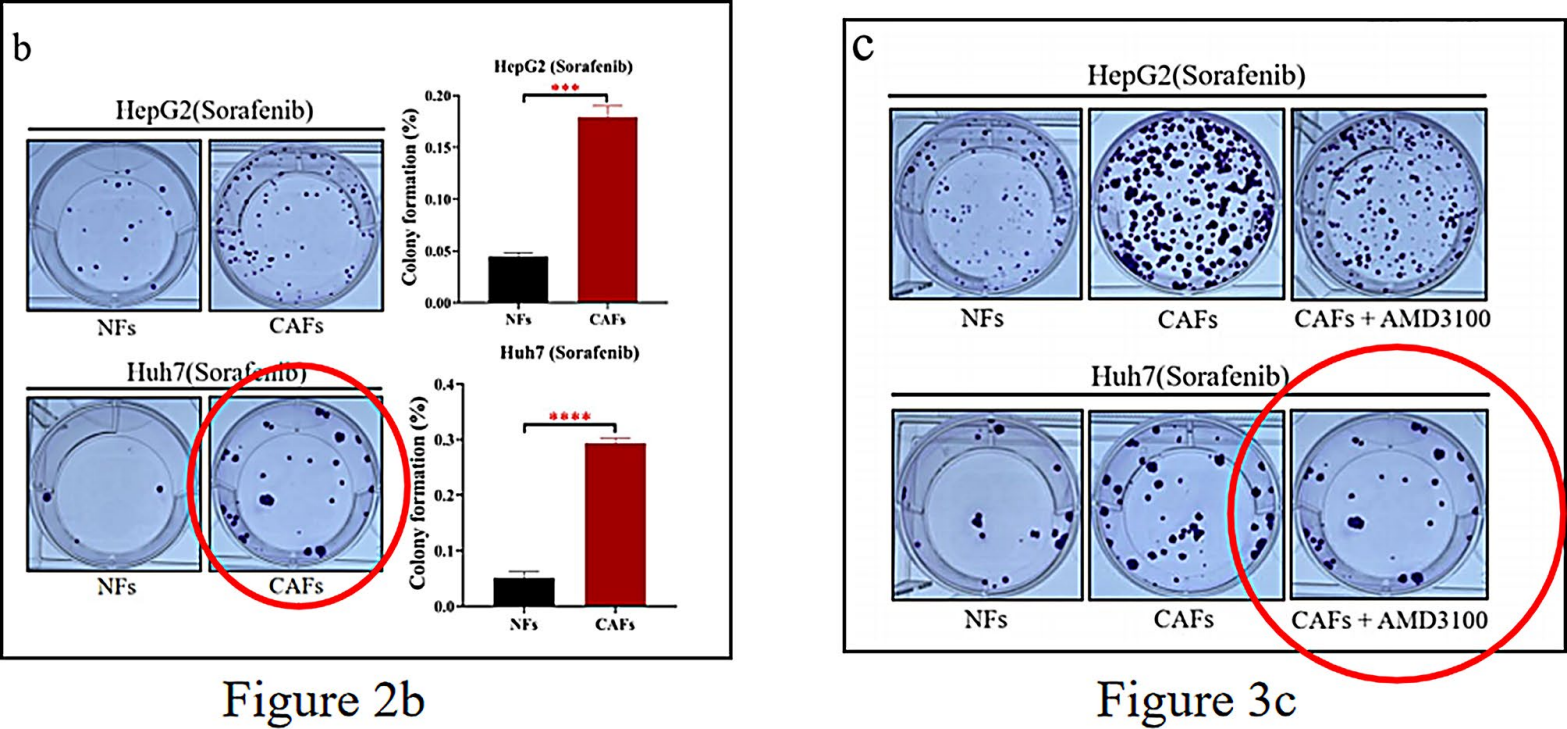


The author sincerely apologizes for the impact this mistake has had on readers. Here is the correct image of “Huh7 (Sorafenib) CAFs” (Figure 2b).

The incorrect Figure 2 is:


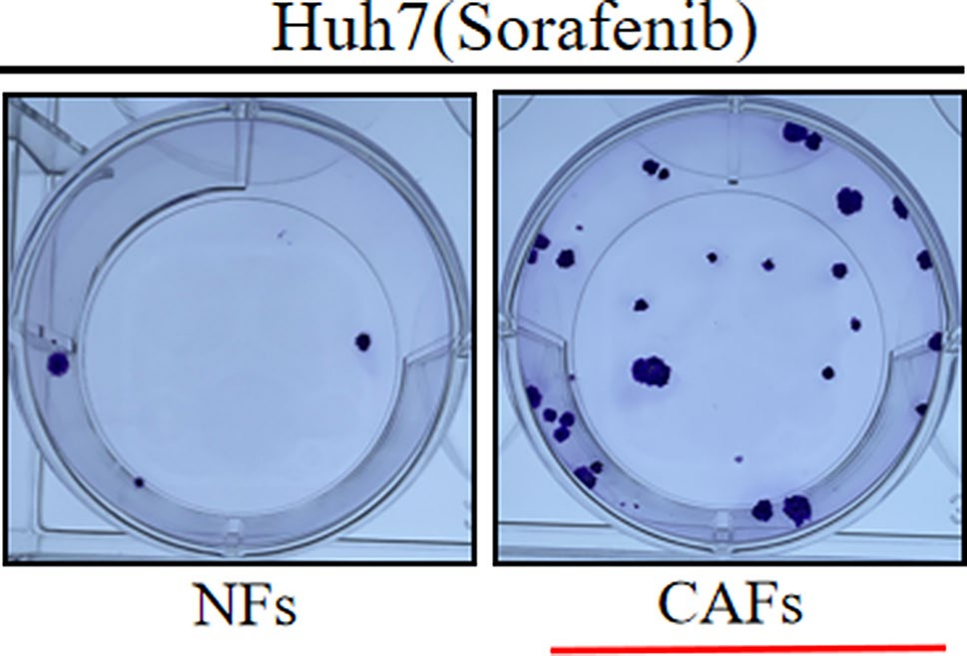


The correct Figure 2 is: