# Supplementary figures and images for: Cancer-associated fibroblasts induce sorafenib resistance of hepatocellular carcinoma cells through CXCL12/FOLR1
Source: BMC Cancer. 2023 Dec 6;23:1198. doi: 10.1186/s12885-023-11613-8 (PMC10701976; doi:10.1186/s12885-023-11613-8)

Figure 3. Full Length blots

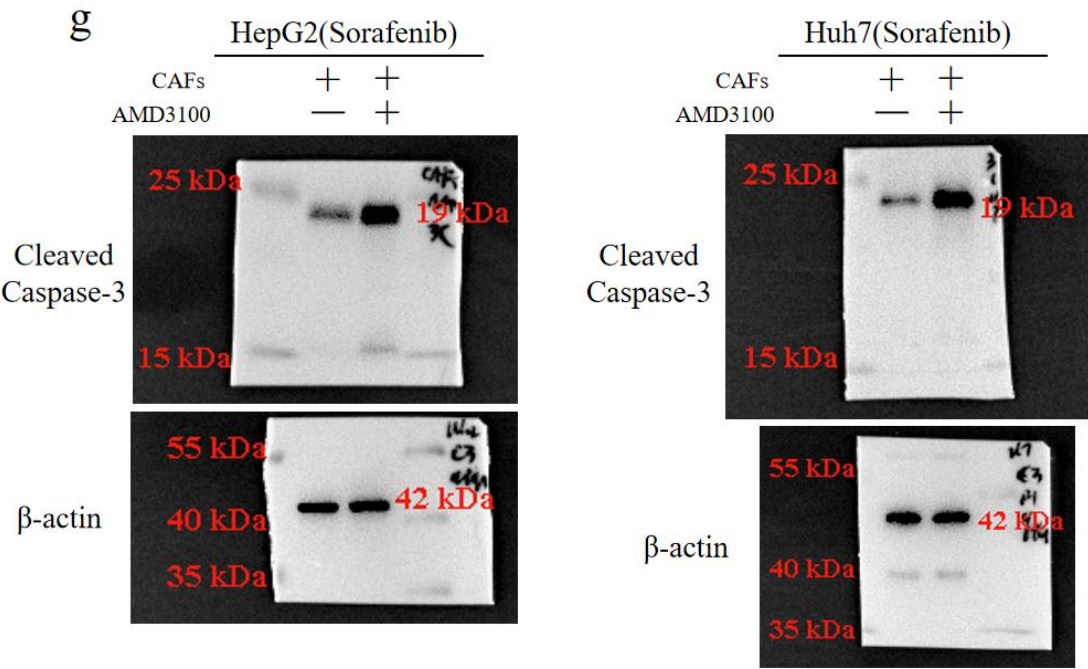

Figure 4. Full Length blots

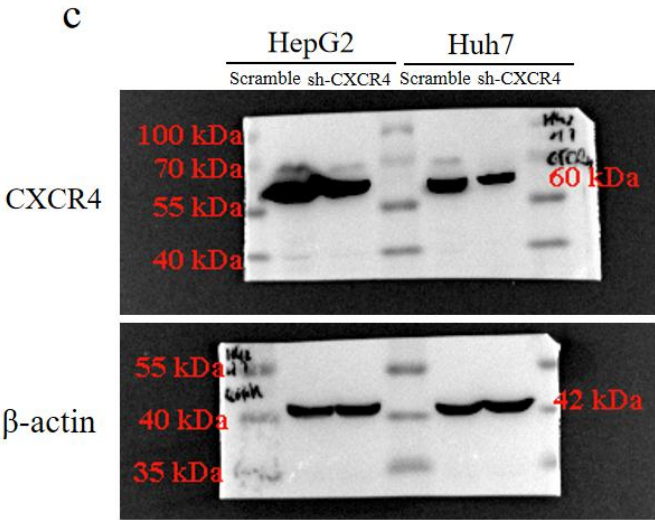

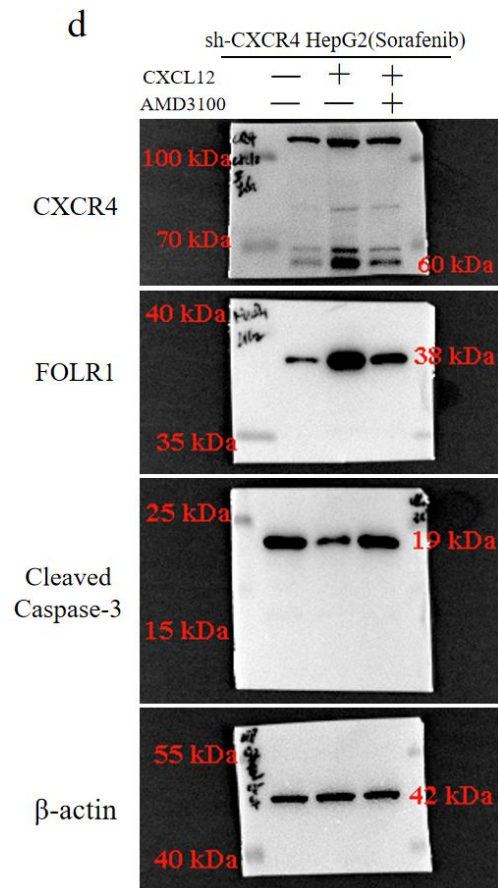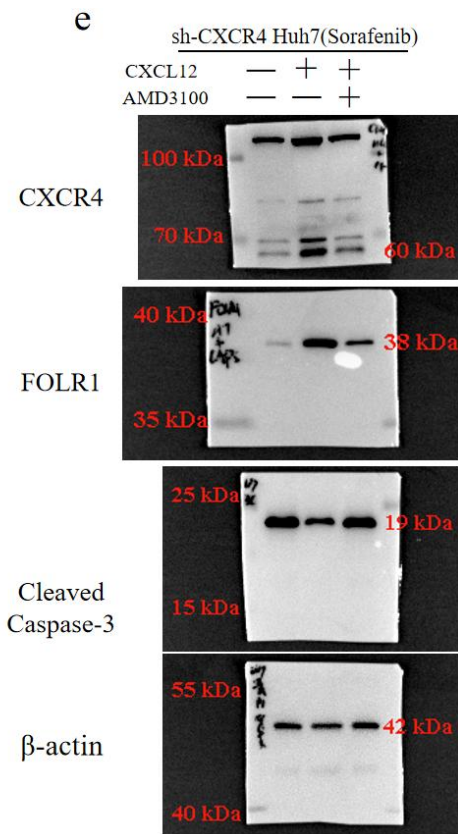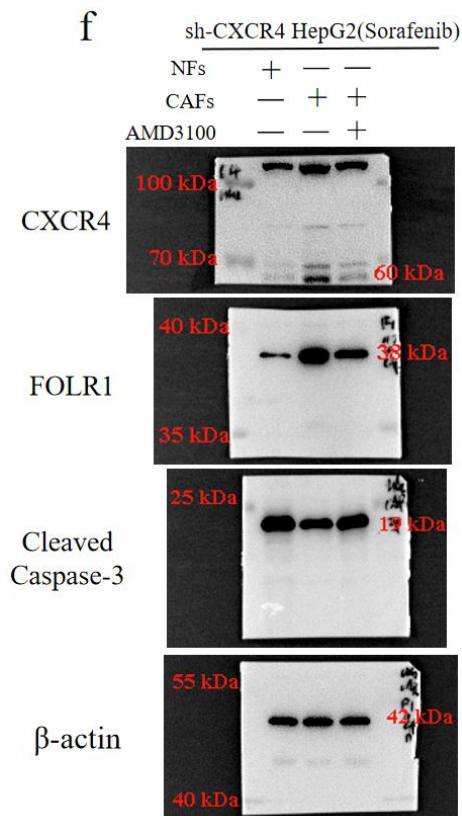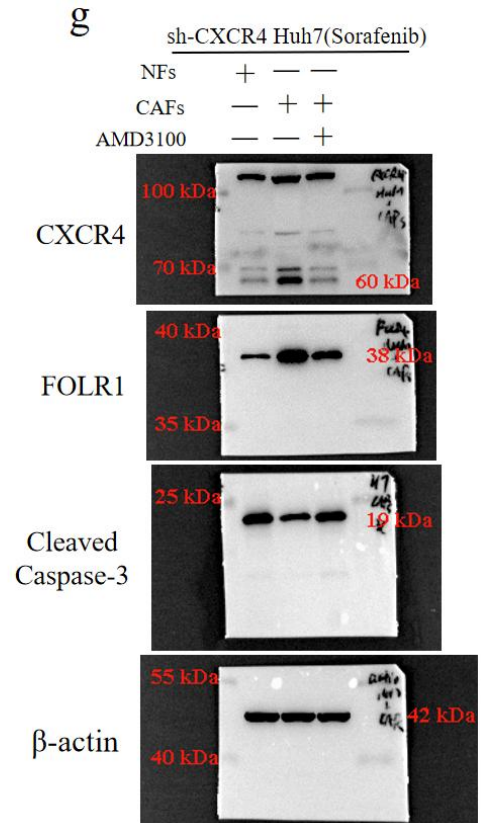

j

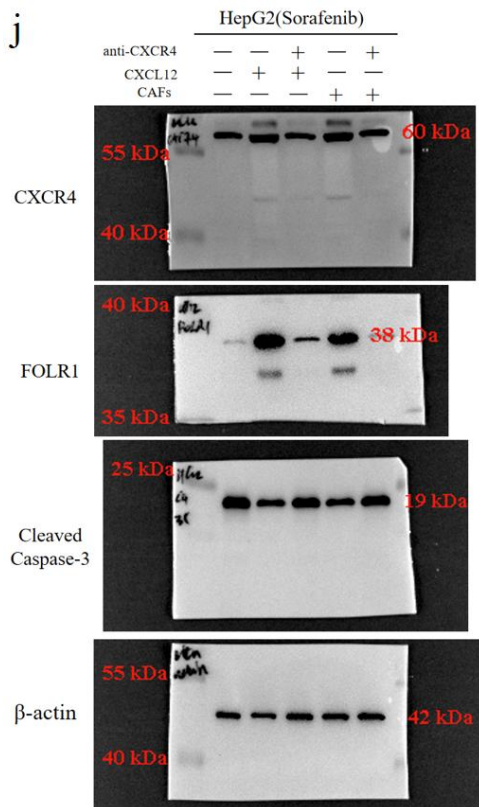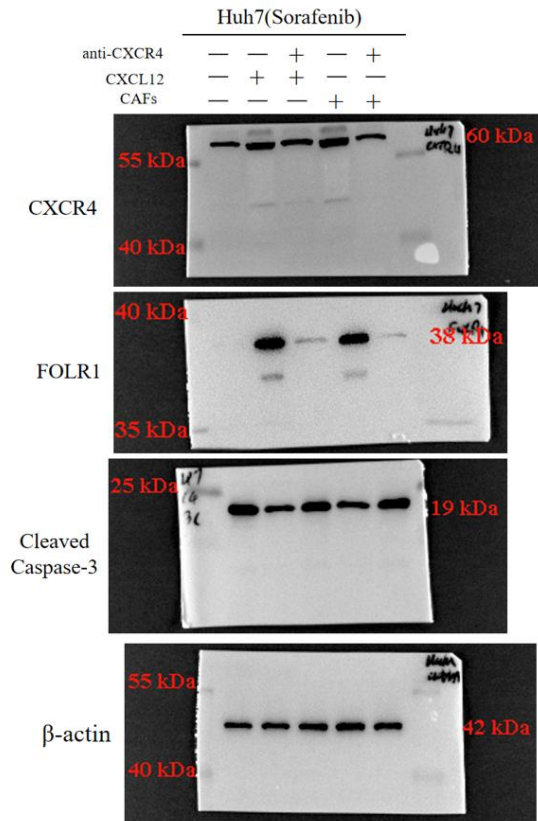

Supplement: Supplementary file 1 — Additional file 1. [file 12885_2023_11613_MOESM1_ESM.pdf]
